# Unilateral diaphragmatic palsy and pleuropericarditis in a patient with granulomatosis with polyangiitis: a case report

**DOI:** 10.1186/s13256-023-04105-7

**Published:** 2023-09-06

**Authors:** Ujjwol Prasad Risal, Raju Prasad Pangeni, Rakshya Pandey, Pradeep Raj Regmi, Aditi Karki, Avish Shah

**Affiliations:** 1 Department of Internal Medicine, Hospital for Advanced Medicine and Surgery, Dhumbarahi, Kathmandu, Nepal; 2 Department of Pulmonology and Sleep Medicine, Hospital for Advanced Medicine and Surgery, Dhumbarahi, Kathmandu, Nepal; 3 Hospital for Advanced Medicine and Surgery, Dhumbarahi, Kathmandu, Nepal; 4 Perfect Diagnostic Centre, Samakhusi, Kathmandu, Nepal; 5 Everest Hospital, New Baneshwor, Kathmandu, Nepal

**Keywords:** GPA, Pleuropericarditis, Unilateral diaphragmatic palsy

## Abstract

**Background:**

Granulomatosis with polyangiitis (GPA) is a rare small vessel vasculitis predominantly affecting upper and lower respiratory tract and kidneys. Unilateral diaphragmatic palsy could be a rare manifestation of GPA. Here we report a case of GPA in a 45-year-old male with unilateral diaphragmatic palsy with pleuropericarditis.

**Case presentation:**

We report a case of a 45-year-old Khas male who presented with acute onset chest pain and shortness of breath who had elevated right hemidiaphragm, bilateral pleural effusion and pericardial effusion who was later diagnosed as GPA.

**Conclusions:**

GPA should be suspected in all patients with diaphragmatic palsy and pleuropericarditis with appropriate clinical and laboratory picture.

## Introduction

Granulomatosis with polyangiitis (GPA) is a necrotizing granulomatous vasculitis predominantly affecting small vessels. It most commonly affects the upper and lower airways and the kidneys. Vasculitic involvement of the phrenic nerve leading to diaphragmatic palsy is extremely rare. Only two cases of unilateral diaphragmatic palsy have been reported in GPA till now [1, 2]. Pleuropericarditis could be another rare manifestation of GPA which has been described in literature [3–6]. Here we describe a case of a 45-year-old male with unilateral diaphragmatic palsy and pleuropericarditis which is the first case with such presentation.

## Case presentation

A 45-year-old Khas male from Kathmandu, Nepal, office-worker by profession, presented to us with complaints of generalized aches and pains all over the body for one week and chest pain for two days. The chest pain was localized to the right lower region, gradual in onset and sharp stabbing in character which was aggravated on deep inspiration. He also complained of dry cough associated with shortness of breath for two days which was increased on exertion. He had no history of fever, rhinorrhea or sore throat or similar history in his family members or colleagues. He had a history of type 2 diabetes mellitus and hypertension for five years. He had no prior history of surgery or trauma. He had been diagnosed as a case of bilateral serous otitis media 20 days prior to this presentation for which bilateral grommet insertion was done at another hospital. However, he had no history of recurrent sinusitis, nasal deformity, hemoptysis, mononeuritis, frothy or bloody urine, skin rash, red eyes, joint pain, Raynaud’s phenomenon, alopecia, photosensitivity, oral or genital ulcers. His current medications included metformin-glimepiride (1gm/2 mg) twice daily, amlodipine-losartan (5/50) once daily and atorvastatin 10 mg once daily. He was a social drinker and non-smoker. He had no significant illness in the family. Physical examination showed tachycardia at 120 bpm, Blood pressure 130/90 mm Hg and respiratory rate 20 per minute with oxygen saturation 90% on room air. Head, ears, eyes, nose and throat (HEENT) examination was unremarkable. Chest examination revealed decreased breath sounds in right infra-scapular and infra-axillary regions. Cardiovascular examination revealed a pericardial friction rub with normal heart sounds. The abdominal, musculoskeletal, nervous system and skin examinations were unremarkable. 

**Fig. 1:**
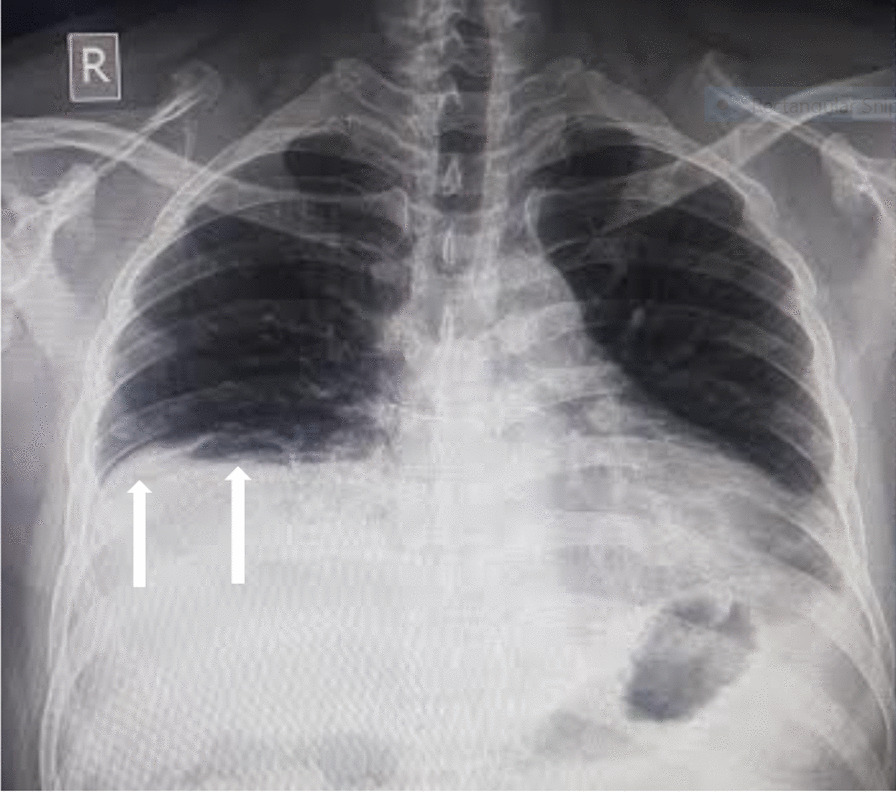
Chest X-ray showing higher level of right hemidiaphragm in comparison to left side (pointed by white arrows). There is also blunting of bilateral costophrenic angles

**Fig. 2:**
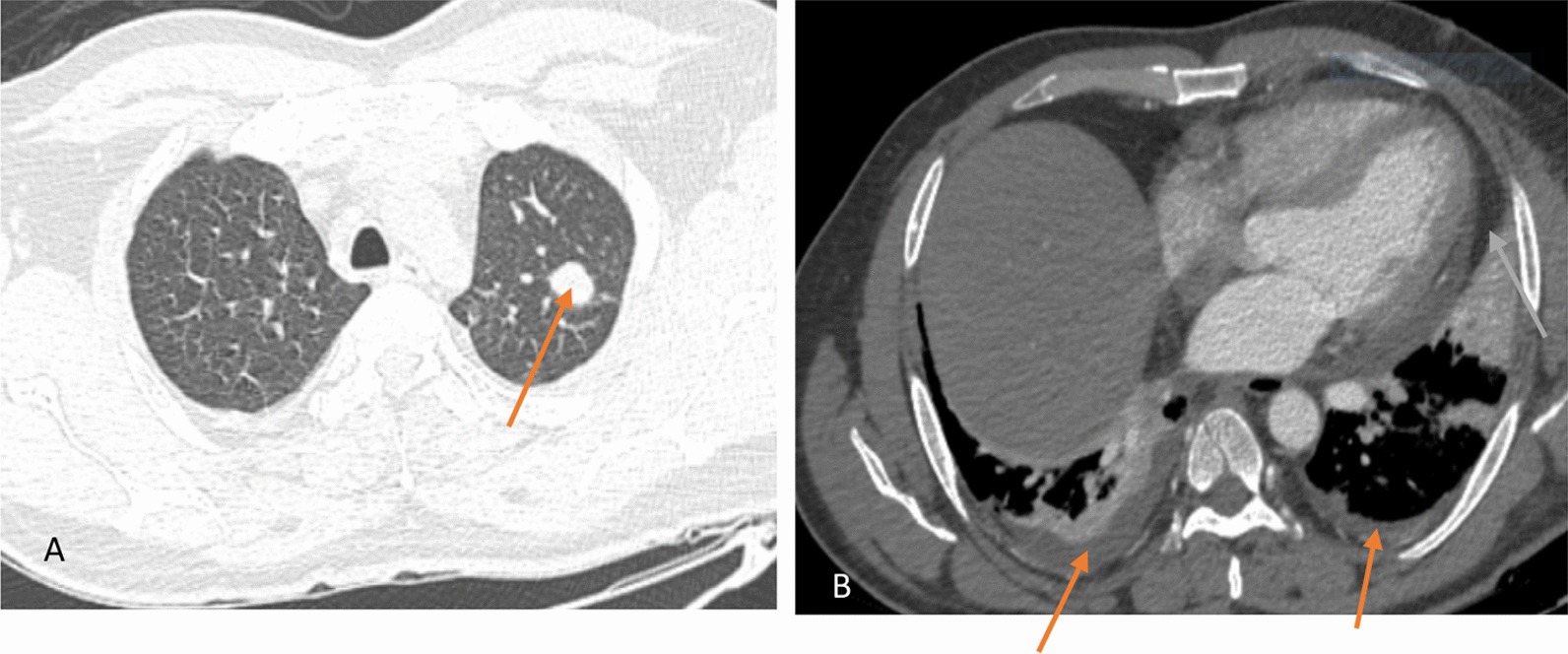
**A** Axial high-resolution CT (HRCT) image shows well-defined nodule in left upper lobe (red arrow). **B** Axial contrast-enhanced CT (CECT) chest shows bilateral pleural effusion (red arrows) and pericardial effusion (white arrow)

His initial laboratory investigations showed mild anemia with neutrophilic leukocytosis (Table 1). His nasal swabs for COVID-19 and influenza were negative. Chest radiography showed elevated right hemidiaphragm with blunting of bilateral costophrenic angles (Fig. 1). Transthoracic echocardiography demonstrated minimal pericardial effusion with normal left ventricle and right ventricle systolic function. The patient was admitted with the working diagnosis of right sided pleuritis with pericarditis. The patient was managed with supportive measures along with empirical antibiotics in the line of pleuro-pericarditis. Further investigations were done including ultrasound of the chest which showed significantly reduced diaphragmatic excursion on the right side. Computed tomography (CT) chest showed a well-defined nodule in left upper lobe with bilateral pleural and pericardial effusion (Fig. 2). Pulmonary function test (PFT) was done which showed significantly improved forced vital capacity in sitting position in comparison to supine position (Table 2). Because of multisystem involvement in the form of bilateral otitis media, phrenic nerve palsy, pleuropericarditis, pulmonary nodule, neutrophilic leukocytosis, raised inflammatory markers, a strong suspicion for GPA was made. Anti proteinase-3(PR-3) was sent which came back strongly positive (Table 1). A final diagnosis of GPA was made and the patient was started on intravenous methylprednisolone pulse of one gram daily for three days. He was also given the first dose of intravenous rituximab at 375 mg/m^2^ which was planned for a total of 4 weeks with prednisolone 60 mg. With the above treatment, his symptoms and laboratory markers started improving. He was discharged after seven days with the final diagnosis of GPA. Till the writing of this report, he had already completed 4 doses of rituximab and he had no further episodes of cough, chest pain, shortness of breath and body ache. His laboratory parameters including C-reactive protein (CRP) had normalized. However, his repeat chest X-ray showed persistent elevation of the right hemidiaphragm.

**Table 1:** Table showing laboratory investigations

	Day 1	Day 4	Day 7	At first follow-up (Day 15)	At second follow-up (after 3 weeks of treatment)
Hemoglobin (gm/dl)	12.4	11.8	12.1	13.9	12.9
White cell count (/mm^3^)	16,510	13,500	17,890	21,460	12,680
Differentials	N78L12E02M08	N80L11M09	N90L04E02M04	N83L06E02M09	N94L04E01M01
Platelets (/mm^3^)	381,000	2,44,000	395,000	238,000	211,000
C-Reactive Protein (mg/L)		82.25	29.49	17.68	2.578
Random Blood Sugar (mg/dl)	155				
Serum creatinine (mg/dl)	0.9	0.72	1.05	1.27	1.14
Serum urea (mg/dl)	22	27	33.9	39	44.2
Serum Sodium (mmol/L)	140	134	138	137	135
Serum Potassium (mmol/L)	3.9	4.3	4.1	4.1	4.6
Serum total bilirubin (mg/dl)	0.2	0.37		0.2	0.25
Serum direct bilirubin (mg/dl)	0.1	0.17		0.12	0.12
Alanine transaminase (ALT) (IU/L)	24	19.4	61	69.2	30.2
Aspartate transaminase (AST)(IU/L)	14	12.3	68	23.3	17.1
Serum total protein (gm/dl)	6.9	6.79		6.56	6.19
Serum albumin (gm/dl)	3.3	3.19		3.57	3.99
Urine RE/ME Albumin 1 + RBC: 3–5/HPF WBC: 4–6/HPF Epithelial cells: 2–3/HPF RBC morphology: 5% dysmorphic RBCs seen					
24-h urinary total protein (mg/24 h)		329.64			

*gm/dl* Gram per deciliter, *mm3* Per cubic millimeter, *mg/L* Milligram per liter, *mmol/L* Millimole per liter, *RBS* Random blood sugar, *ALT* Alanine transaminase, *AST* Aspartate transaminase, *IU/L* International unit per liter, *RE/ME* Routine examination, microscopic examination, *RBC* Red blood cell, *WBC* white blood cell, *mg/24* hr milligrams per 24 hours

**Table 2:** Table showing pulmonary function test

	% Predicted (supine)	% Predicted (sitting)
Forced vital capacity (FVC)	45	58
Forced expiratory volume in 1 second (FEV1)	44	56
FEV1/FVC	98	96

## Discussion

Granulomatosis with polyangiitis (GPA), formerly called Wegener’s granulomatosis, is a rare immunologically mediated systemic disease of unknown etiology whose hallmark features include necrotizing granulomas and pauci-immune vasculitis that most commonly affects the upper respiratory tract, lungs and the kidneys [7]. There are many causes of acute onset dyspnea in GPA that include sub-glottic stenosis, diffuse alveolar hemorrhage, pericarditis, which are related to the direct effects of the disease on the respiratory tract and/or the heart. Unilateral diaphragmatic palsy can cause acute dyspnea as it can reduce breathing capacity by more than a third [8]. Till now, only two cases of unilateral diaphragmatic palsy have been reported in GPA, one of which was an incidental finding [1]. The mechanism of phrenic nerve palsy in GPA is due to inflammation of vasa nervorum of the phrenic nerve leading to axonal ischemia [9]. Other causes of phrenic nerve palsy like trauma, tumor and aortic aneurysm were ruled out in our patient.

The most common pleural manifestation in GPA is pleural effusion which can be primary or secondary to renal failure [10]. Similarly, pericarditis is the most common cardiac manifestation in GPA [3]. In one study 88 of 1058 patients (8.3%) had pleuritis and/or pericarditis as a manifestation of vasculitis [11]. Our patient had right sided pleuritis and pericarditis with asymptomatic effusion on the left. Other causes of effusion such as heart failure, renal failure, hypoalbuminemia and hypothyroidism had been ruled out. The mechanism of pleuropericarditis could be related to vasculitis of the vessels supplying the pericardium and pleura [12]. Our patient had a score of 8 (cut-off score of 5) based on the latest classification criteria for GPA [13]. This is the first documented case of GPA manifesting with pleuropericarditis and unilateral diaphragmatic palsy. There are no clear-cut guidelines for the treatment of phrenic nerve palsy in GPA. However, assuming the same mechanism of injury as in mononeuritis multiplex and also because of pleuropericarditis, induction with high dose steroid and rituximab was done as per the latest guidelines [14]. Our patient showed improvement in both symptoms and laboratory parameters but no improvement in diaphragmatic palsy as indicated by persistent raised right hemidiaphragm. It remains to be seen if his diaphragmatic palsy will revert to normal.

## Conclusions

Unilateral diaphragmatic palsy could be a sole neurological manifestation of GPA. Pleuropericarditis in a patient with multi-system involvement should raise the suspicion for GPA.

## Data Availability

Not applicable.
